# Supplementary material for: Targeting netrin‐3 in small cell lung cancer and neuroblastoma
Source: EMBO Mol Med. 2021 Mar 15;13(4):e12878. doi: 10.15252/emmm.202012878 (PMC8033513; doi:10.15252/emmm.202012878)
Supplement: Supplementary file 1 — Expanded View Figures PDF [file EMMM-13-e12878-s001.pdf]

Expanded View Figures

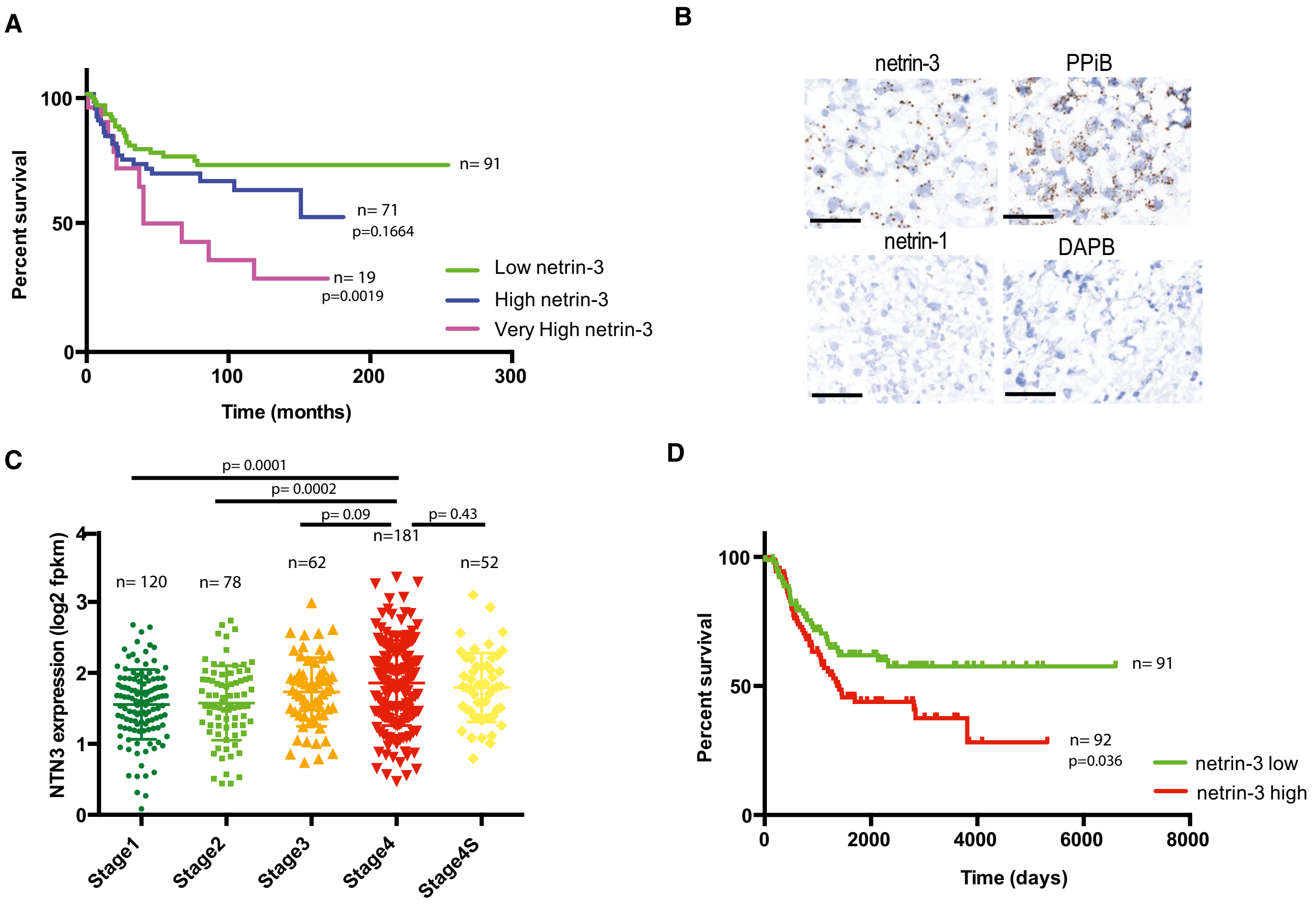

Figure EV1. Netrin-3 role in NB.

A Netrin-3 high expression is a marker of poor prognosis in NB. 280 months overall Kaplan–Meier survival curves in a panel of 181 patients of all NB stages. The cohort was dichotomized base on netrin-3 median expression as Fig 2C (in green). An additional group (purple) defined as netrin-3 very high, expressing twice the median expression. High netrin-3 is the group composed by the patient of the high group of Fig 2C, without the very high group of patients. Statistical treatment of the data: Mantel–Cox; *P*-value is indicated below the graph.

B Representative netrin-3 and netrin-1 detection, using RNAscope on NB high-grade tumor cryosection. Negative control DAPB, positive control PPiB. Each brown dot represents a unique molecule of mRNA of each target gene. Scale bars 20 μm.

C Quantification of netrin-3 expression by RNA sequencing in a panel of 498 human NB stages 1, 2, 3, 4, and 4S. The number of cases is indicated on the graph. Statistical treatment of the data was performed using a two-sided Student's *t*-test with Welsh correction (bars indicate SD).

D Netrin-3 high expression is a marker of poor prognosis in aggressive NB. 6,000 days overall Kaplan–Meier survival curves in a panel of 183 stage 4 patients (extracted from the 498 RNA-seq cohort). The cohort was dichotomized base on netrin-3 expression.

© 2021 The Authors

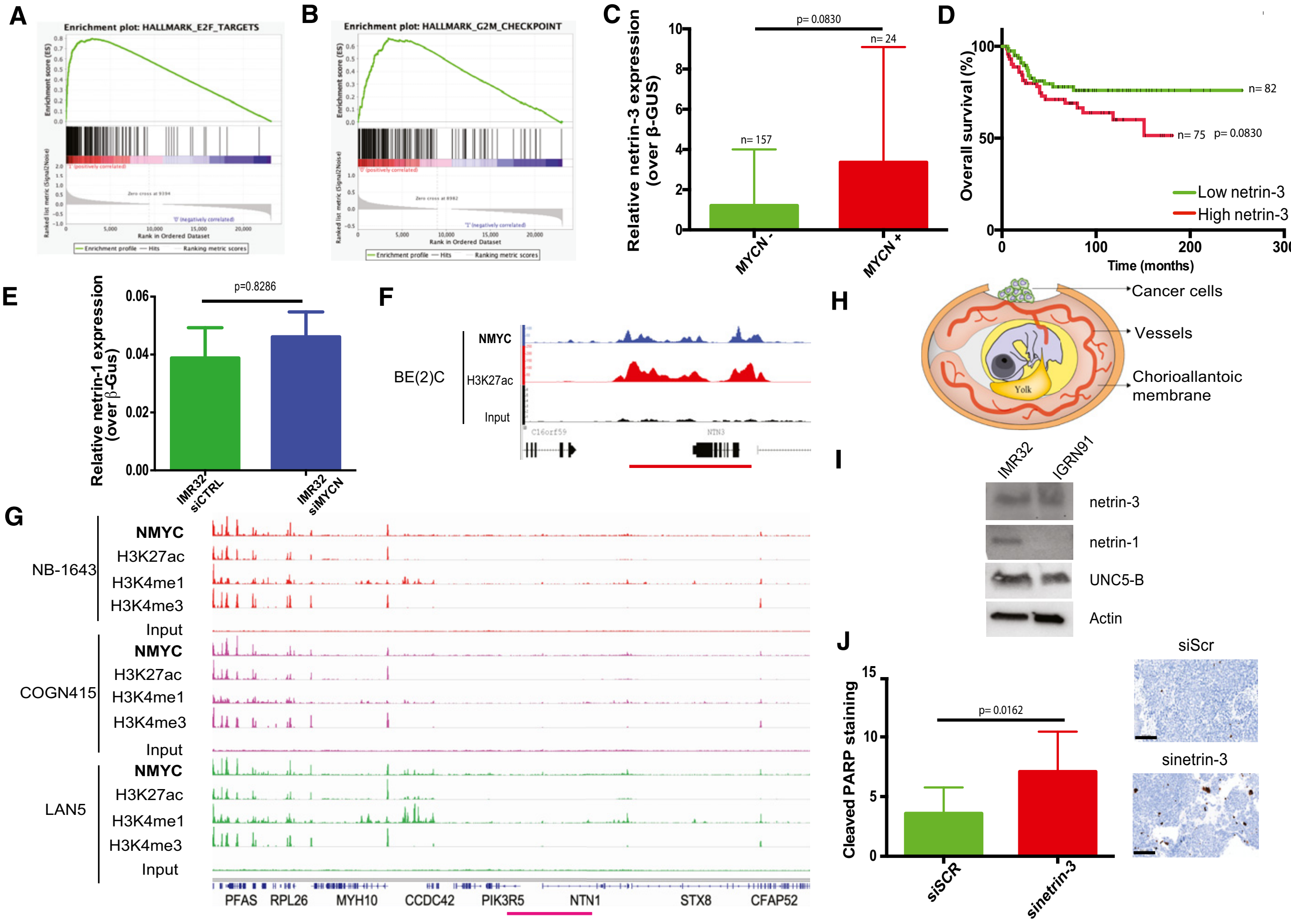

Figure EV2. Netrin-3 is a target gene of MYCN in NB.

A Gene Set Enrichment Analysis (GSEA) in high netrin-3 NB patients. The total cohort was dichotomized with the $n = 50$ lowest vs. 50 highest: amplification of E2F pathway ($P \leq 0.001$; fdqr = 0.0014).

B Gene Set Enrichment Analysis (GSEA) realized in stage 4 patients high vs. low netrin-3 NB patients ($n = 183$ characterized in EV-1C). The hallmarks G2/M checkpoint is amplified, indicating a strong proliferation in the High netrin-3 group ($P = 0.025$; fdqr = 0.044).

C Analysis of netrin-3 expression in MYCN-amplified NB patients. The cohort was dichotomized based on MYCN amplification and sorted for netrin-3 expression. Statistical treatment of the data: Welsh test; *P*-value is indicated on the graph ($n = 181$, bars indicate SD).

D Analysis of patient survival based on netrin-3 expression on patient none MYCN-amplified from the cohort described in Figs 2A and Fig EV2A ($n = 157$). Statistical treatment of the data: Mantel–Cox; *P*-value is indicated below the graph.

E QRT–PCR analysis of *netrin-1* gene expression in IMR32 ($n = 4$) and IGRN-91 (not shown; *netrin-1* not detectable) cell line after MYCN silencing by siRNA (*U*-test, bars indicate s.e.m.).

F Analysis of MYCN binding on netrin-3 promoter in BE2(C) NB cell line after Chip-seq experiment. MYCN peaks upstream of netrin-3 are associated with a broad H3K27ac-enriched region.

G ChIP-seq analysis of MYCN binding, active enhancer epigenetic marks H3K27ac, H3K4me1, and active promoter epigenetic mark H3K4me3, on *netrin-1* locus (pink line). An enrichment of MYCN, associated with active enhancer marks, was detected in three different neuroblastoma cell lines NB-1643, COGN415, and LAN5.

H Schematic representation of the experimental chick chorioallantoic membrane (CAM) model. IMR32 or IGR-N91 cells were transiently transfected with scramble, netrin-1 and netrin-3 siRNAs and xenografted on CAM on day 10.

I NB cell lines were subjected to immunoblots.

J Representative picture of cleaved PARP staining. Quantification of IGR-N91 cells positive for cleaved PARP, in tumors transfected or not by siNetrin-3 or siControl ($n = 4$, *U*-test, bars indicate SD, scale bars 100 μm).

© 2021 The Authors

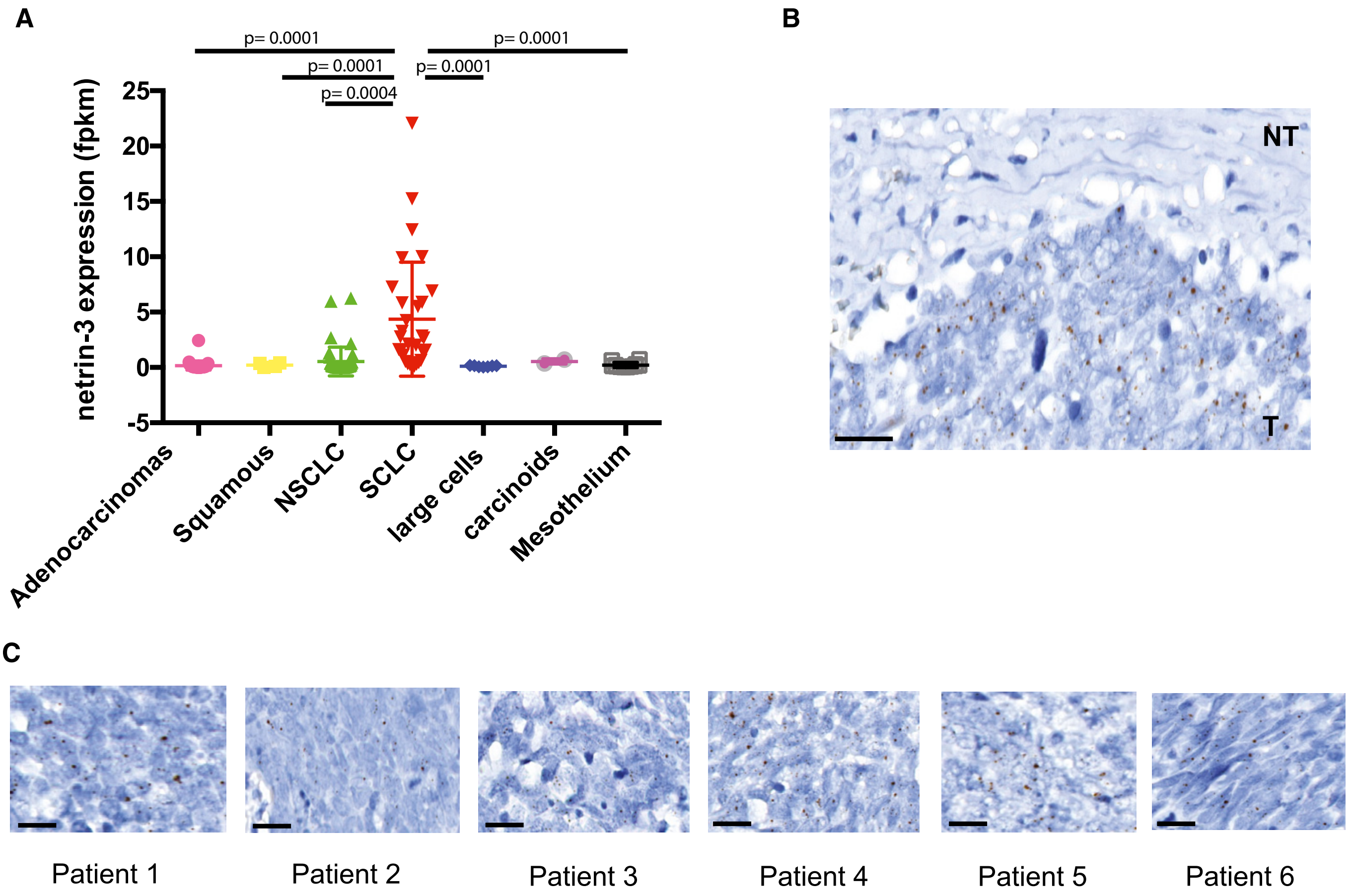

Figure EV3. Netrin-3 function in SCLC.

A Quantification of netrin-3 expression by RNA sequencing in lung cancer cell lines. Statistical treatment of the data was performed using a two-sided Student's *t*-test with Welsh correction (adenocarcinomas $n = 36$; squamous $n = 4$, NSCLC $n = 47$, SCLC $n = 30$, large cells $n = 7$, carcinoids $n = 2$, mesothelium $n = 12$; bars indicate SD).

B Netrin-3 is not expressed in the tumor microenvironment. Representative netrin-3 mRNA detection, using RNAscope technology on SCLC paraffin-embedded tumor sections. T = tumoral part; NT = not tumoral. Each brown dot is a unique molecule of mRNA of netrin-3 in the tumoral part. Scale bars 20 µm.

C Representative netrin-3 mRNA detection, using RNAscope technology on SCLC paraffin-embedded tumor sections. Each brown dot is a unique molecule of mRNA of netrin-3 in the tumoral part. Scale bars 20 µm.

© 2021 The Authors

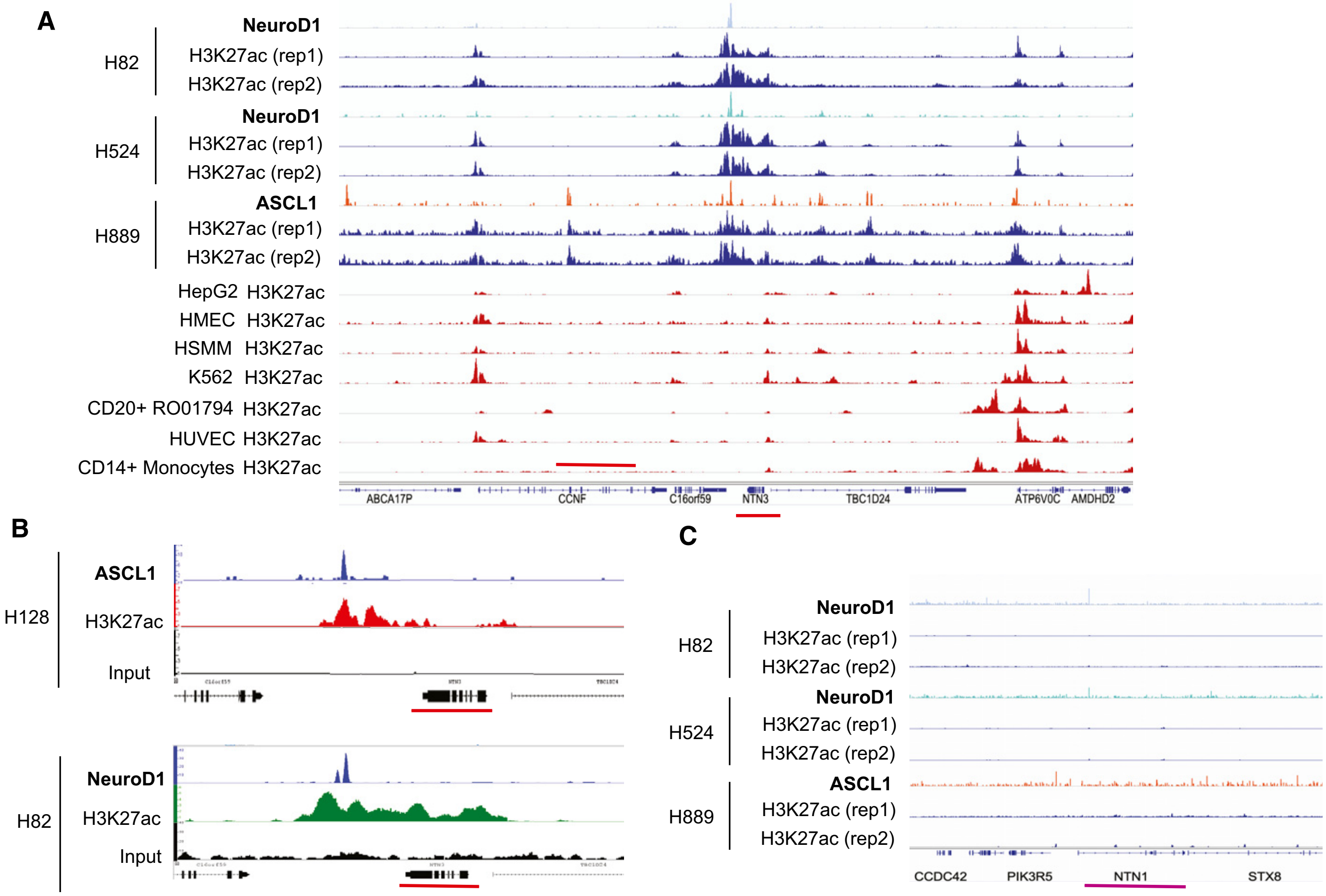

Figure EV4. Netrin-3 is a target gene of NeuroD1 and ASCL1 in SCLC.

A Analysis of NeuroD1 ChIP-sequencing in two cell lines (light blue), and ASCL1 ChIP-sequencing data in three cell lines (light red) along with input controls for SCLC cell lines expressing either NeuroD1 or ASCL1 (black) on *netrin-3* gene promoter (red line). H3K27ac-enriched region is specific of SCLC cell lines.

B Analysis of NeuroD1 or ASCL1 and H3K7ac recruitment to the netrin-3 promoter region in two SCLC cell lines.

C Analysis of NeuroD1 ChIP-sequencing in two cell lines (light blue), and ASCL1 ChIP-sequencing data in one cell line (light red) along with input controls for SCLC cell lines expressing either NeuroD1 or ASCL1 (black). On *netrin-1* gene promoter (pink line).

© 2021 The Authors

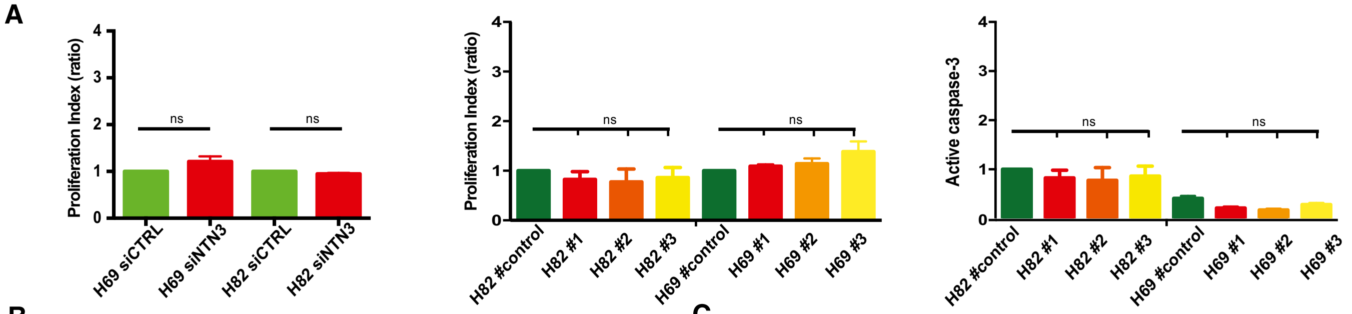

B

Epitope NP-137 (23AA)
netrin-1 ARRCRFNMELYKLSGRKSGGVC

		Identities
netrin-3	20/22	90.91%
netrin-4	10/22	45.45%
netrin-5	17/22	77.27%
netrin-G1	3/22	14.29%
netrin-G2	2/22	9.09%

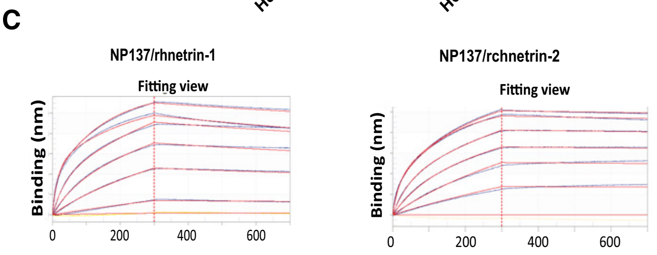

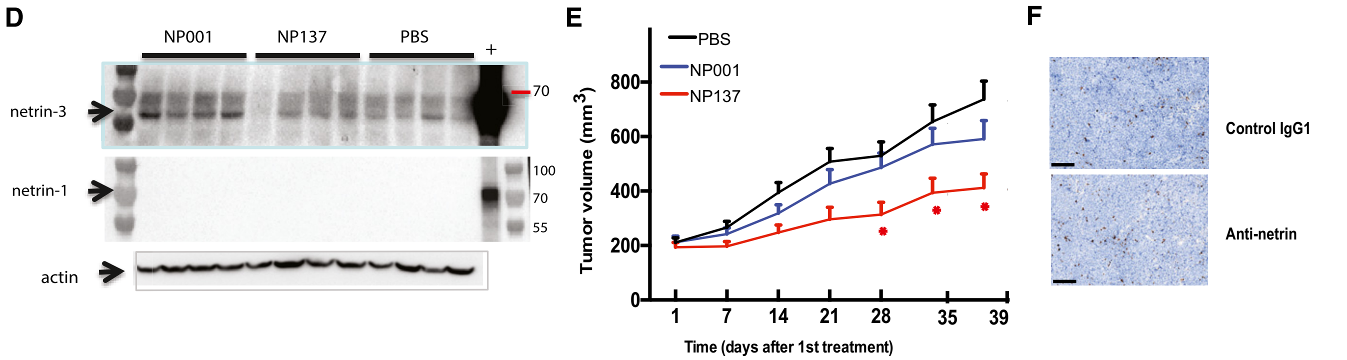

Figure EV5. Netrin-3 as a therapeutic target.

A Proliferation and cell death assay analyses H82 and H69 transfected with siNetrin-3 and in CRISPR/Cas9 clones lacking netrin-3 in NCI-82 and NCI-H69. Proliferation was determined by the MTT method; apoptosis by cleaved caspase-3 monitoring. (Error bars indicate SD, $n = 4$, *U*-test).

B Table presenting the percentage of conservation of the NP137 epitope across netrin family members.

C Analysis of NP137 binding in a dose-dependent manner on netrin-1 and netrin-2L by bio-layer interferometry assays.

D NMRI *nude* mice were engrafted with NCI-H82 cells by subcutaneous injection of 2 million cells. When the mean tumor volume reached approximately 80 mm^3, animals were treated 3 × time/weekly by intra-abdominal injection of PBS; NP001 (Human IgG1, isotype); NP137 (anti-netrin1/3) for 20 days. Immunoblots of netrin-1 and netrin-3 were conducted at end point with the corresponding antibodies.

E NMRI *nude* mice were engrafted with NCI-H2286 cells by subcutaneous injection of 5 million cells. When the mean tumor volume reached approximately 80–100 mm^3, animals were treated 3 × time/weekly by intra-abdominal injection of PBS; NP001 (Human IgG1, isotype); NP137 (anti-netrin1/3) for 20 days. $n = 10$ animals/group (*$P < 0.05$, two-way ANOVA, Error bars indicate s.e.m.).

F Representative images of cleaved PARP staining in H82 treated with NP001 (Human IgG1, isotype) or NP137 (anti-netrin1/3). Scale bars 100 μm.
