# Supplementary figures and images for: Targeting netrin‐3 in small cell lung cancer and neuroblastoma
Source: EMBO Mol Med. 2021 Mar 15;13(4):e12878. doi: 10.15252/emmm.202012878 (PMC8033513; doi:10.15252/emmm.202012878)

Expanded View 2I

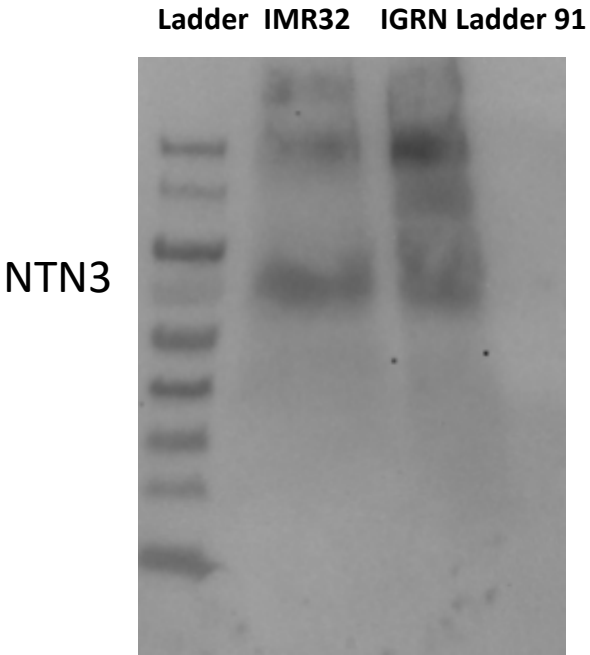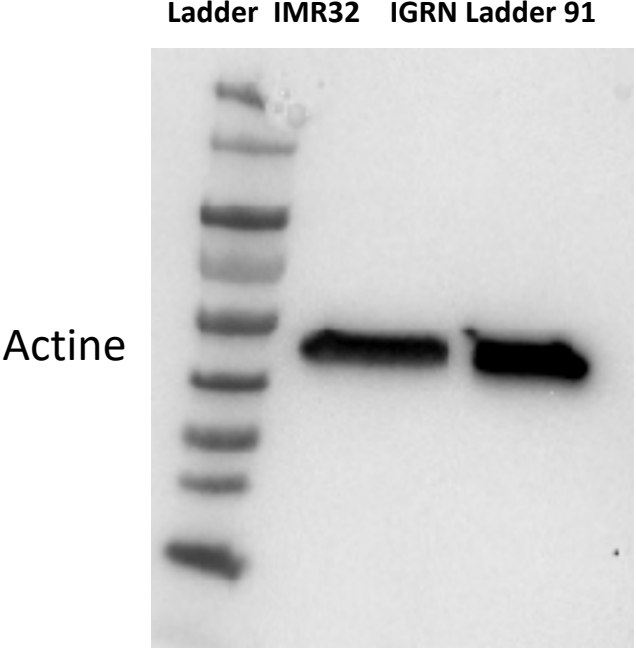

Expanded View 2I

netrin 1

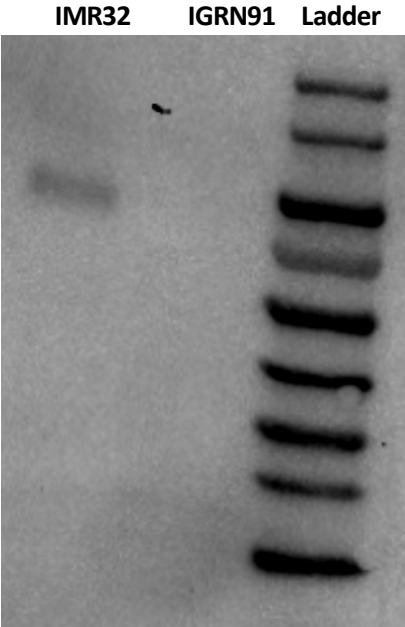

UNC5B

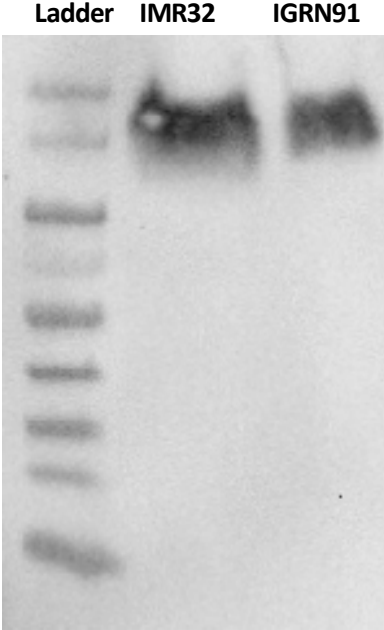

Supplement: Supplementary file 2 — Source Data for Expanded View [file EMMM-13-e12878-s004.zip › EMM-2020-12878-V3-EV2_Figure_Source_Data-sd.pdf]

Expanded view 5D

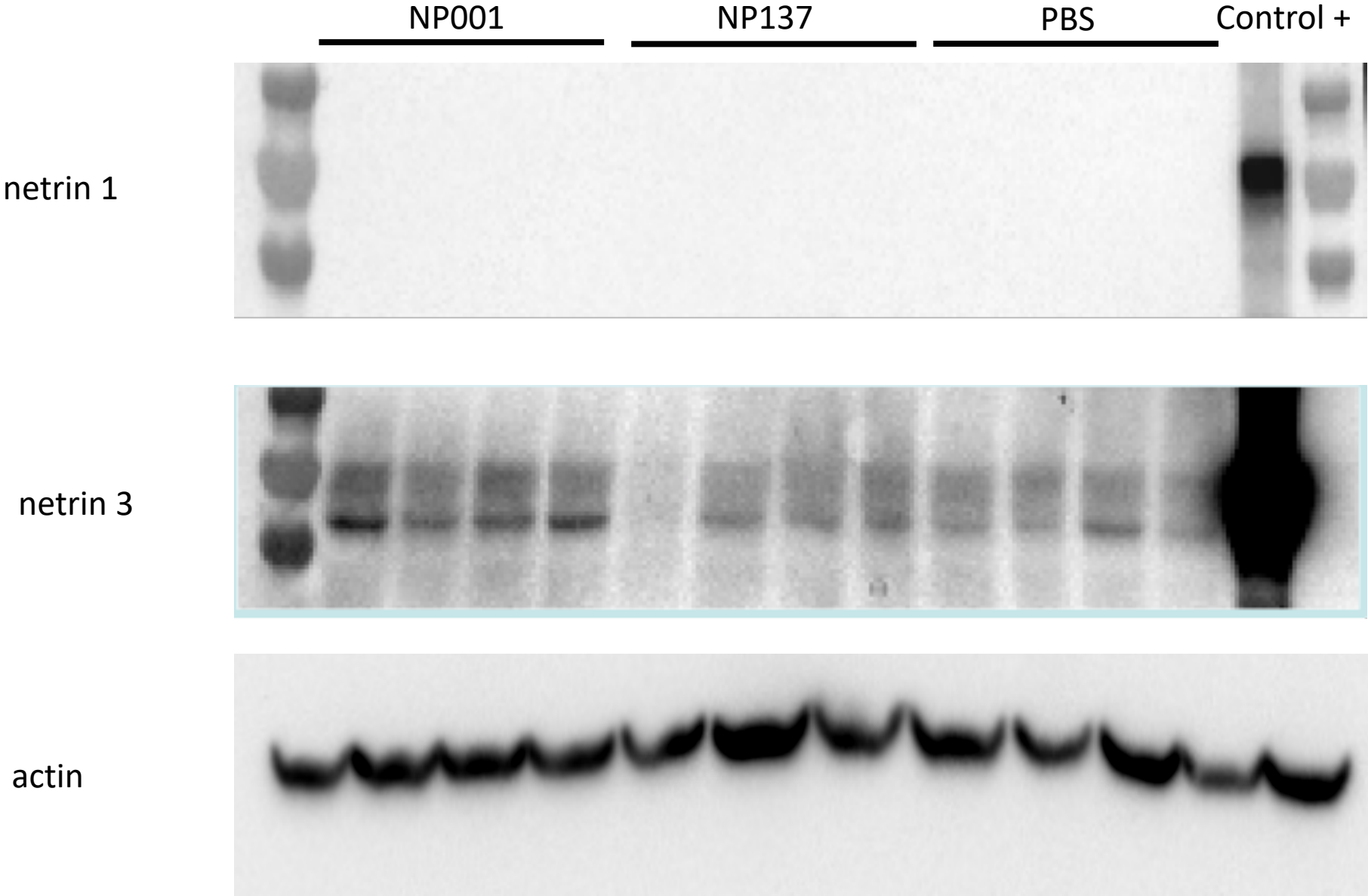

Supplement: Supplementary file 2 — Source Data for Expanded View [file EMMM-13-e12878-s004.zip › EMM-2020-12878-V3-EV5_Figure_Source_Data-sd.pdf]
