# Supplementary material for: Targeting netrin‐3 in small cell lung cancer and neuroblastoma
Source: EMBO Mol Med. 2021 Mar 15;13(4):e12878. doi: 10.15252/emmm.202012878 (PMC8033513; doi:10.15252/emmm.202012878)

Figure 6C

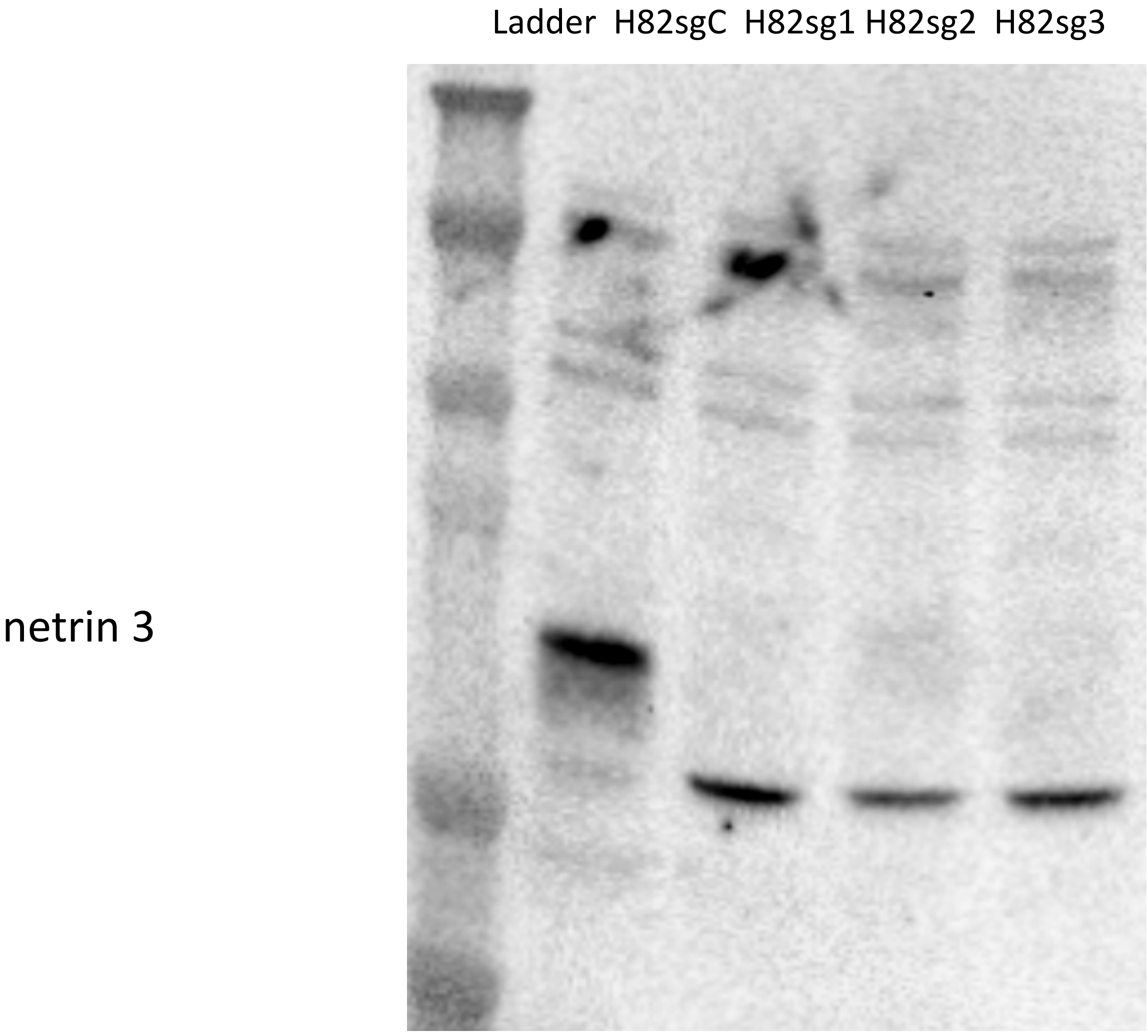

Figure 6C

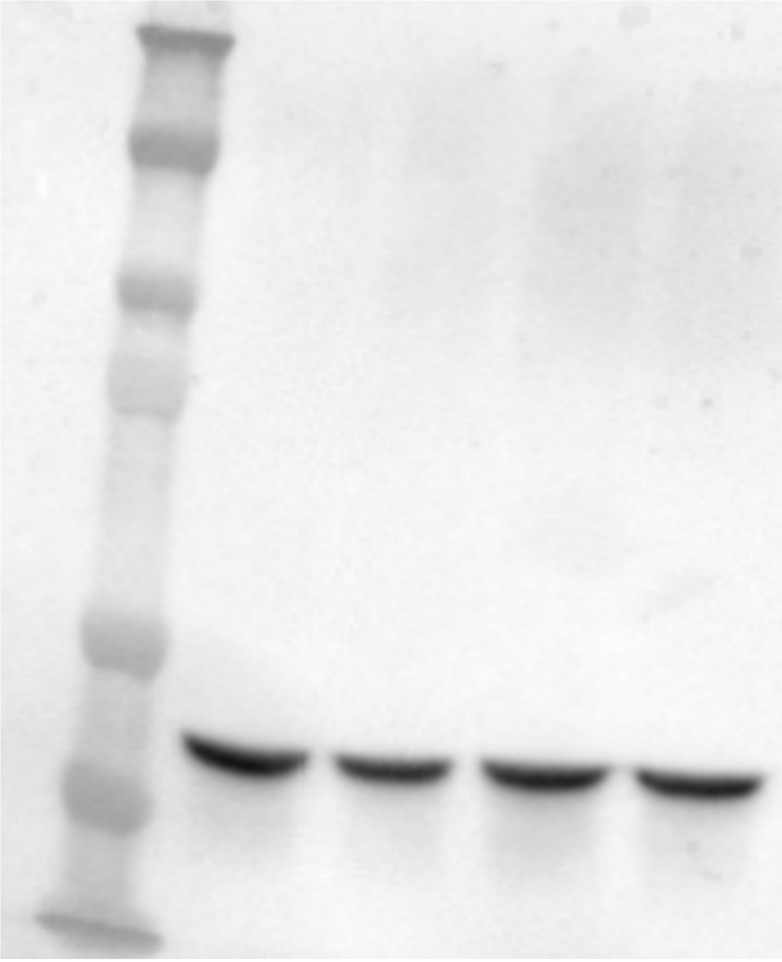

Figure 6C

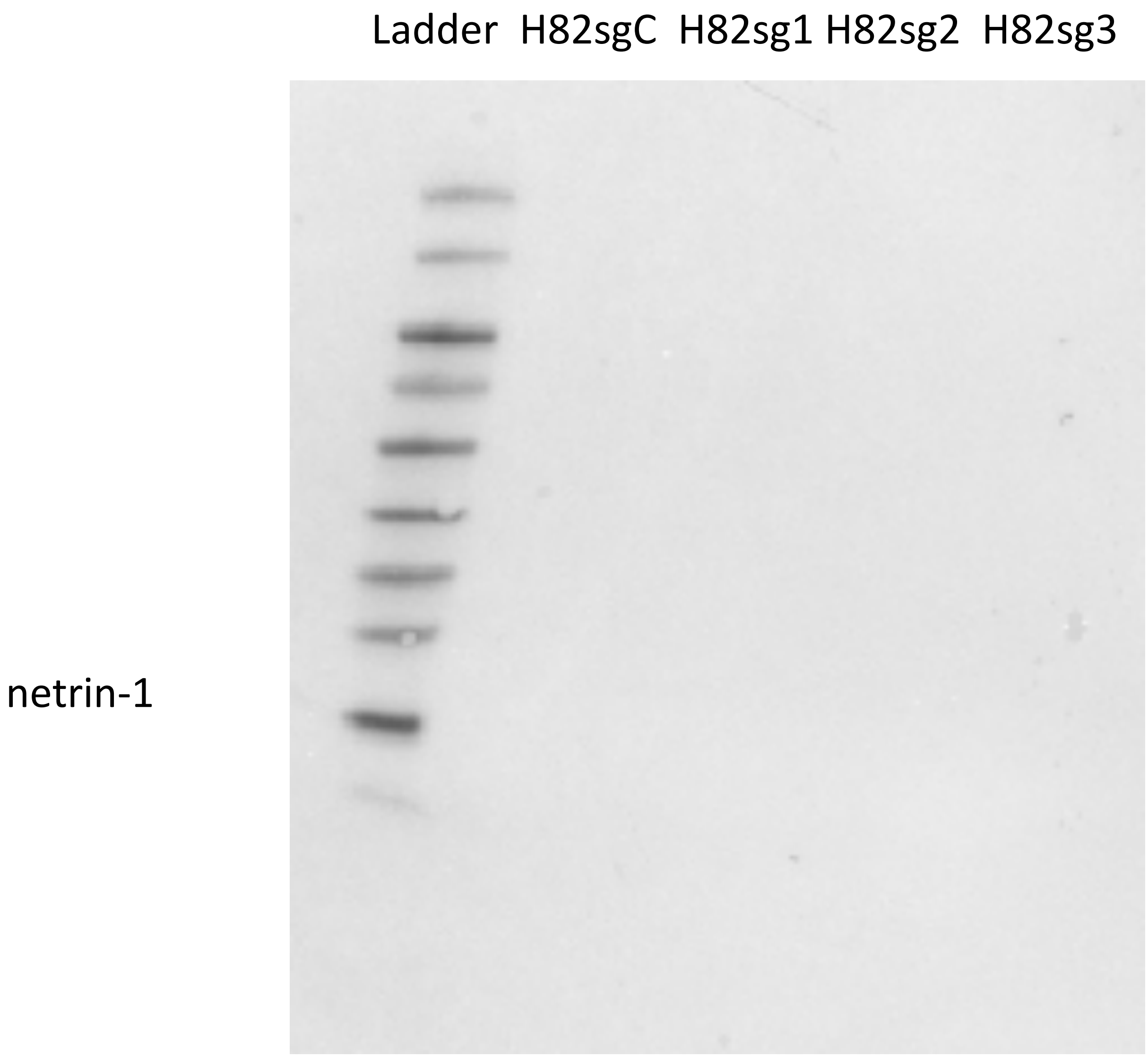

Figure 6C

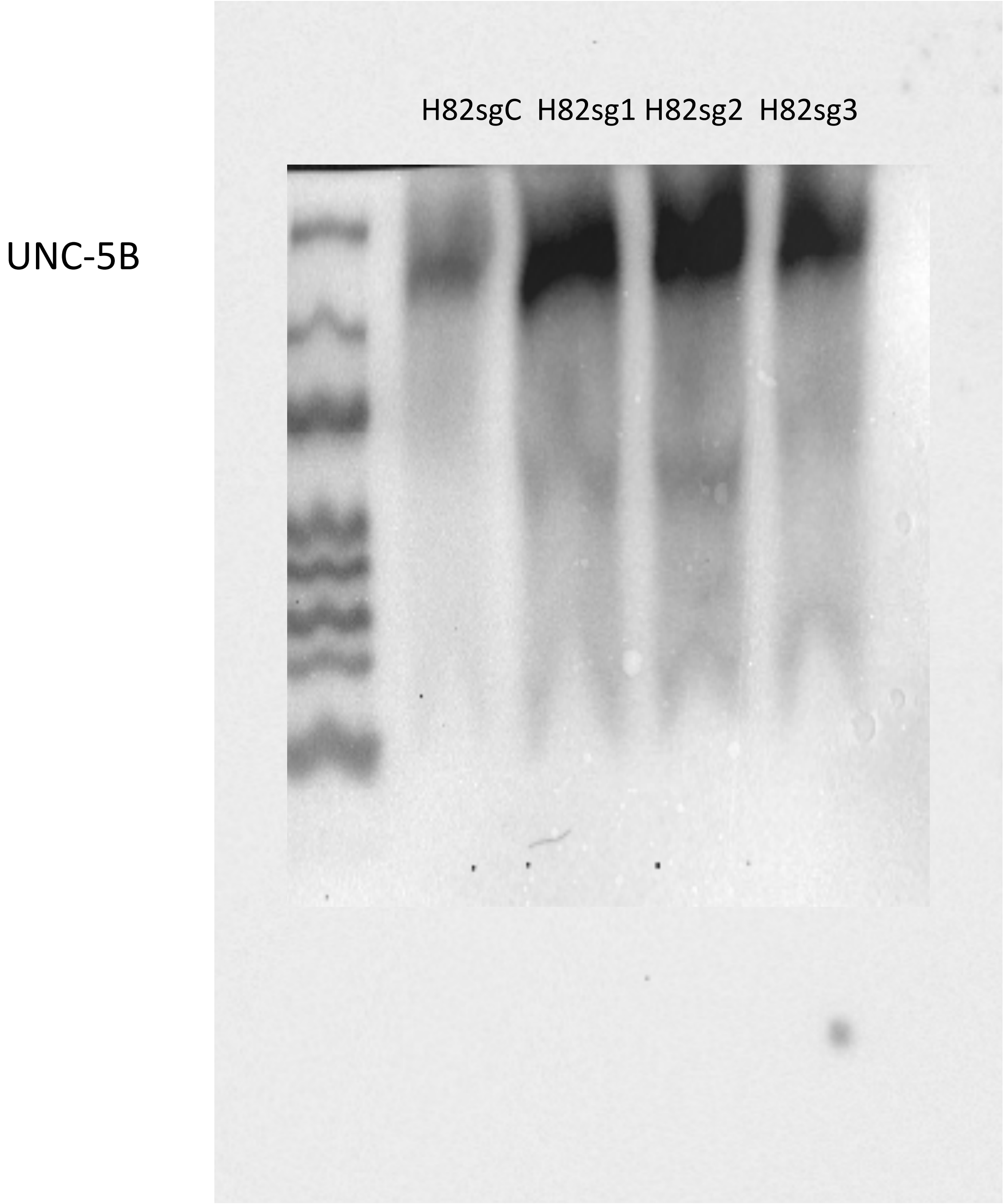

Figure 6C

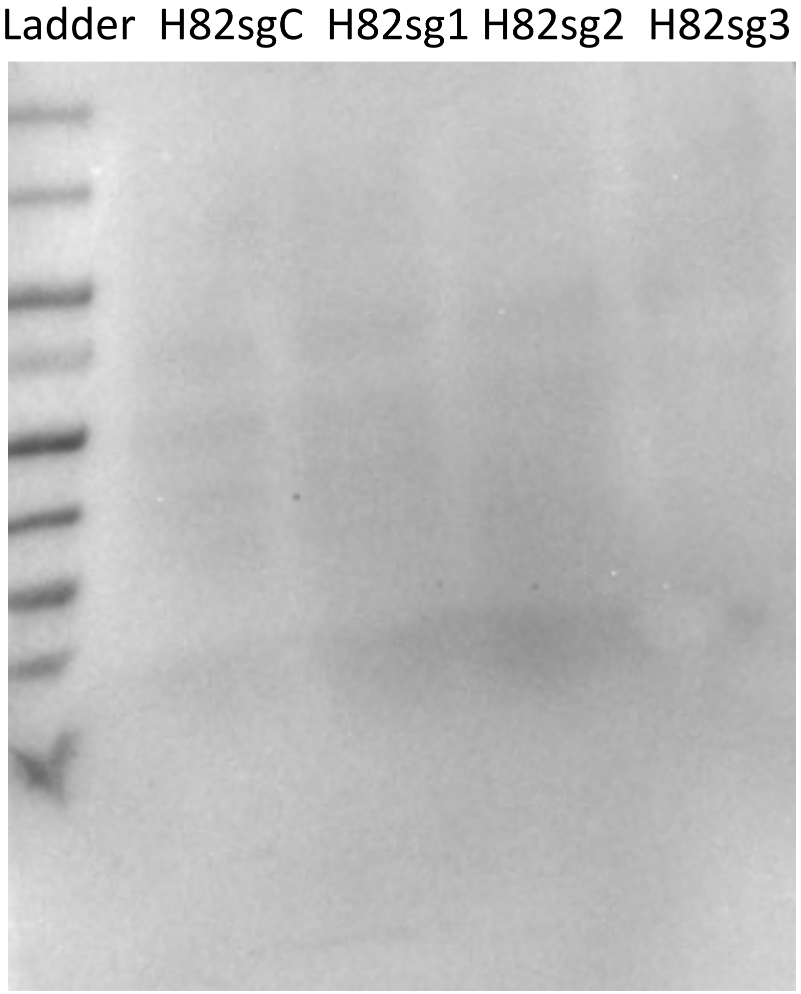

unc 5C

Figure 6E

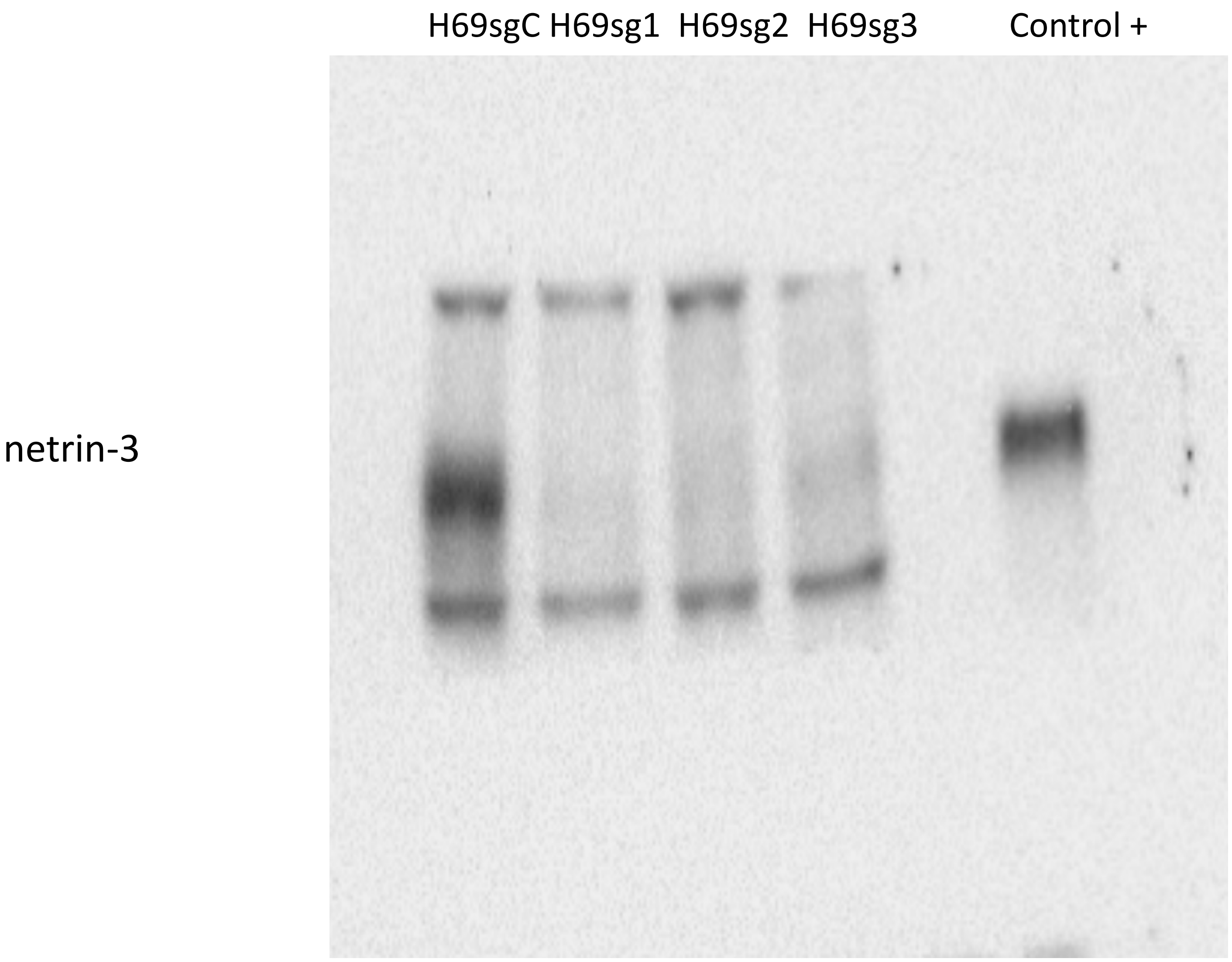

Figure 6E

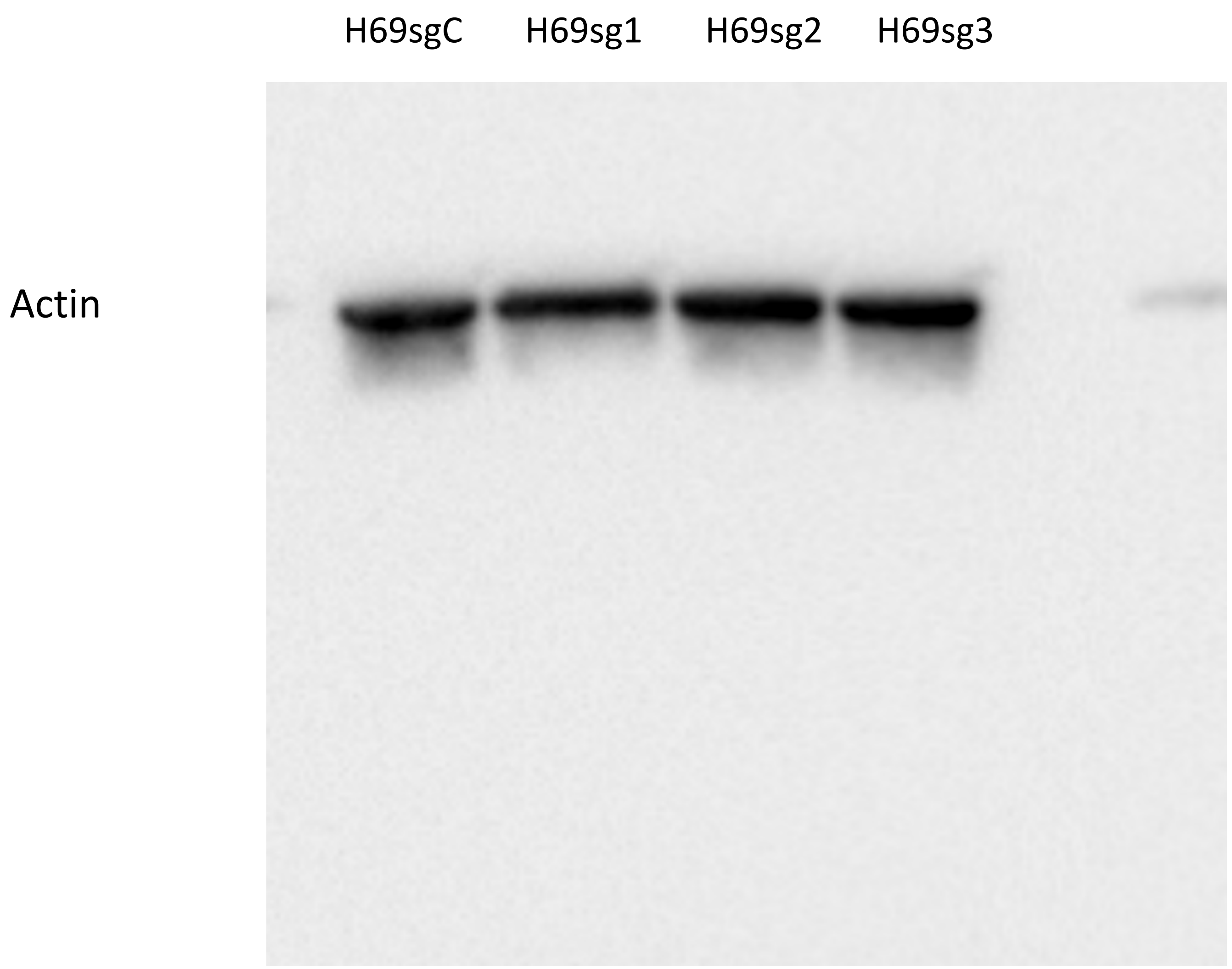

Figure 6E

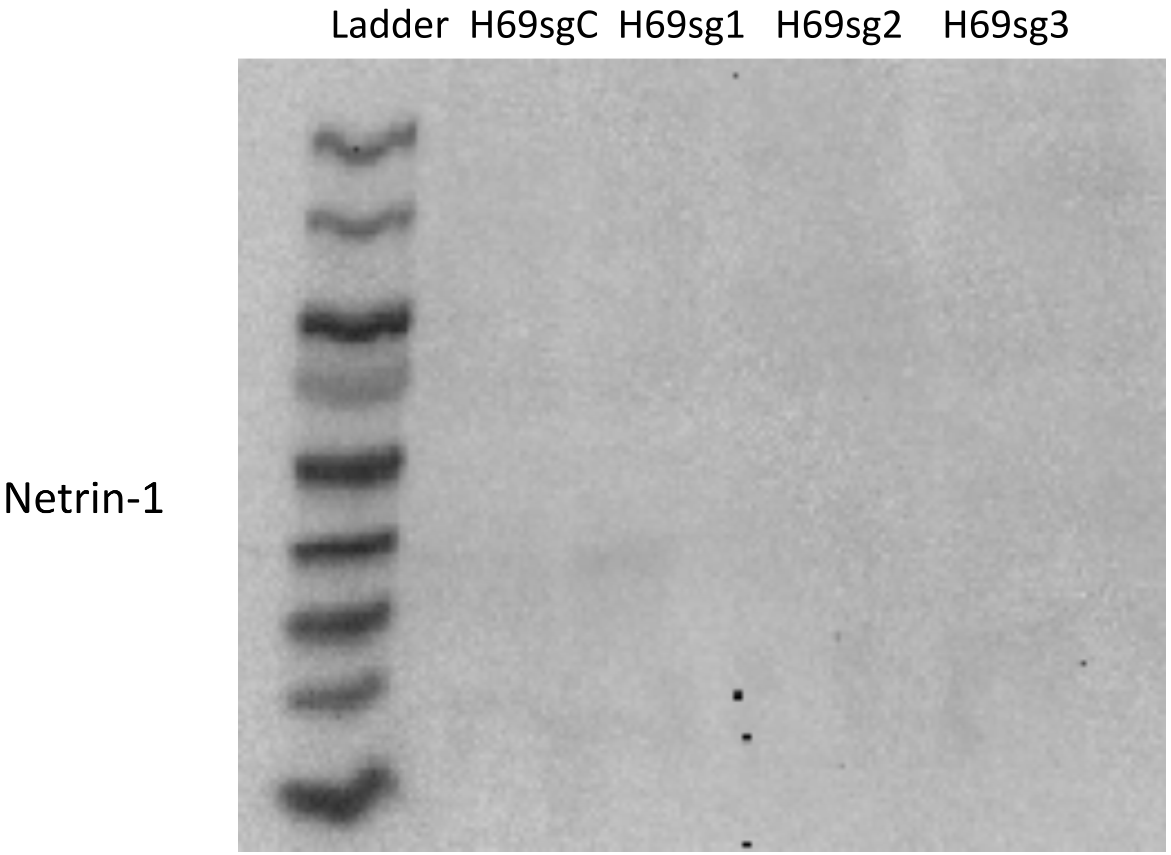

Figure 6E

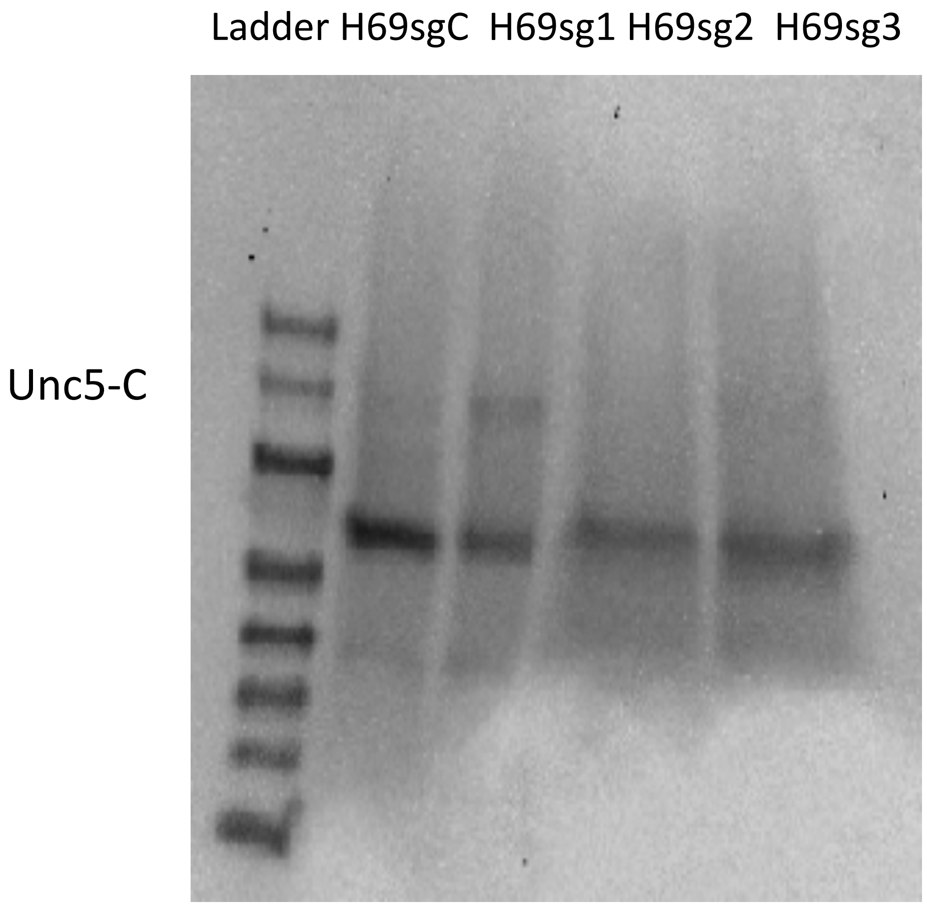

Figure 6E

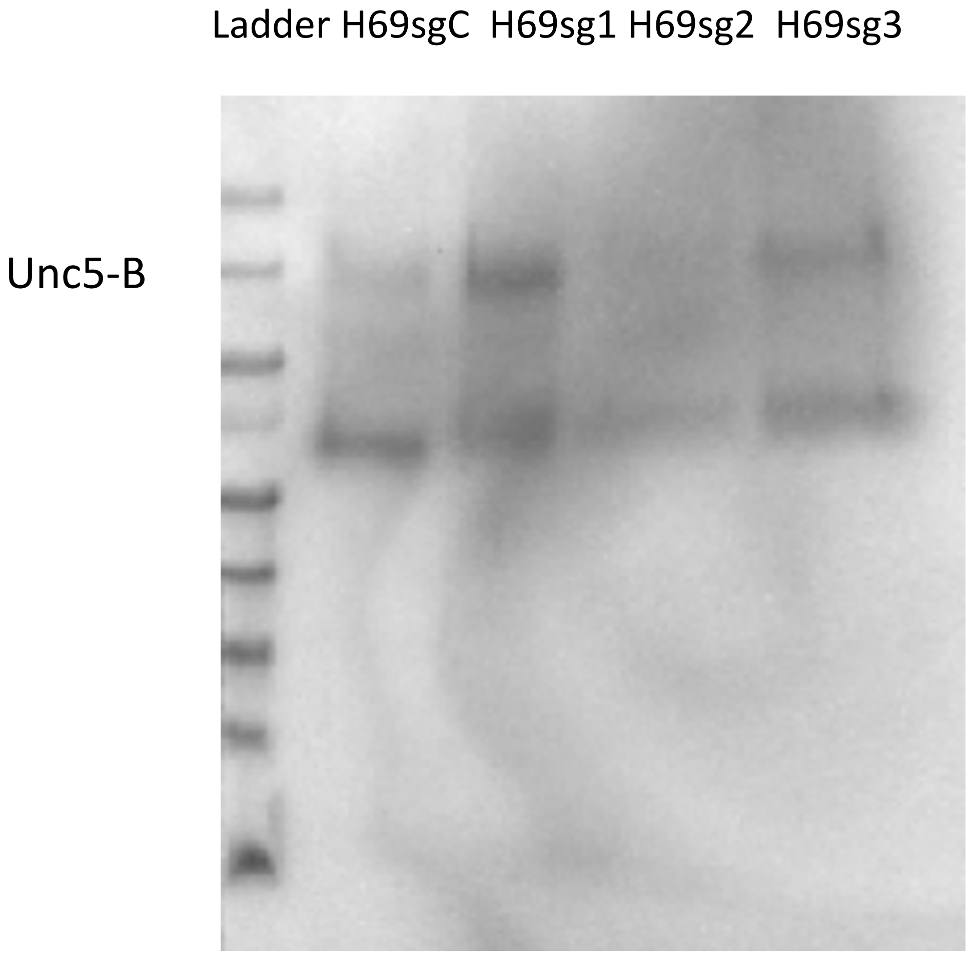

Supplement: Supplementary file 4 — Source Data for Figure 6 [file EMMM-13-e12878-s003.pdf]
